# In-home work environment for home care workers in Northern Sweden before and during the Covid-19 pandemic

**DOI:** 10.1186/s12913-024-12161-y

**Published:** 2025-01-24

**Authors:** Fredrik Norström, Karin Bölenius, Klas-Göran Sahlén, Magnus Zingmark, Anita Pettersson-Strömbäck

**Affiliations:** 1 Department of Epidemiology and Global Health, Umeå University, Umeå, 901 87 Sweden; 2 Department of Nursing, Umeå University, Umeå, Sweden; 3 Health and Social Care Administration, Östersund, Sweden; 4 Department of Community and Rehabilitation, Umeå University, Umeå, Sweden; 5 Department of Psychology, Umeå University, Umeå, Sweden

**Keywords:** Staff, Equipment, Work conditions, Cross-sectional study

## Abstract

**Background:**

The in-home work environment is the main work environment for home care workers, but it has only been sparsely studied. Our aim was to investigate the in-home work environment for home care workers by exploring challenges that arise regardless of a pandemic and by investigating Covid-19–specific challenges.

**Methods:**

Two cross-sectional studies were conducted, one before (2017) and one during the pandemic (2021/2022) in three Swedish regions (Jämtland/Härjedalen, Västerbotten and Västernorrland), in which 1,154 (58%) out of 2,000 and 629 (33%) of 1,900 invited home care workers participated, respectively. Participants responded to a questionnaire asking about 10 problems associated with the in-home work environment as well as Covid-19–related challenges. Comparisons were conducted between regions and between study years using univariable analyses.

**Results:**

Daily problems with the in-home work environment were common before the pandemic, and they increased statistically significantly during the pandemic for, among other things, non-ergonomic beds (29% vs. 37%), impractical bathrooms (40% vs. 50%), indoor smoking (24% vs. 31%), and pets (19% vs. 25%). There were major concerns about the risk of getting infected with Covid-19 for both staff (42%) and the home care recipients (50%). There were statistically significant differences between regions, e.g. many problems were more common in the Västerbotten region than in the other two regions during the pandemic, while challenges with protective equipment was most common in the Västernorrland region.

**Conclusions:**

In-home work environment problems are common for home care workers and worsen in a more strained situation. Efforts are needed to strengthen the work environment for home care workers.

**Supplementary Information:**

The online version contains supplementary material available at 10.1186/s12913-024-12161-y.

## Background

Home care workers in Sweden spend most of their time in the homes of those receiving care. Their work environment and well-being are thus influenced by interactions with care recipients and others, including relatives to the care recipient, as well as the available equipment and the condition of the home. Although this is a key factor in the work environment of home care workers, we are aware of only one study that has examined how the in-home work environment affects these workers [1]. In that Norwegian qualitative study, challenges with verbal abuse, stressful work conditions, second-hand smoking, poor sanitary conditions, and ergonomic limitations were identified.

Home care services in Sweden focus mainly on providing personal care for the home care recipient and performing household chores [2]. These services are mainly financed by taxes, but also by user fees from the recipient, which at most are 235 euros per month in 2024 [3]. Home care service is provided after an individual application and a formal needs assessment by a care manager in accordance with the Social Services Act. While municipalities have the responsibility of the delivery and quality of this support, it can also be provided by private companies that are financed by the municipality. According to the work environment act [4], the employer is responsible for preventing physical, psychological and social risks for employees, and due to this the municipality may make improvements such as replacing the bed in the homes of the home care recipients. Home care workers are represented by a mixture of assistant nurses, nursing assistants, and staff with no formal health education. Most assistant nurses have two to three years of education at the high school level, which is the main difference compared to nursing assistants, who have a more limited health care education [5, 6].

The work environment for home care workers is not only affected by the in-home work environment, but is also affected by the overall workload of the staff. Home care services have during the last few decades seen a large increase in the number of home care recipients that home care workers meet on a daily basis, from an average of 4.0 patients in the 1980s [7], to 8.6 persons a day in 2005, and to 11.8 recipients a day in 2015 [8]. This increase has been reported to make it challenging to complete work tasks with as much as 40% of home care workers experiencing too much to do [8]. Challenges with high workload among home care workers have been reported not only in Sweden [9–11], but also in Norway and Switzerland [12, 13], and there is a connection between high workload and negative health consequences and rising sick leave rates within the profession [10, 12, 14, 15]. Considering that a third to a half of the home care workers in Nordic countries are considering quitting, with the highest rates reported in Sweden [16, 17], a good in-home work environment should be seen as an important component for lower staff turnover.

During the initial stages of the Covid-19 pandemic, Sweden was one of the countries most affected by the spread of the disease, at least in regard to reported mortality rates [18]. The high mortality rates were to a large extent related to the virus being spread within nursing homes, where there were reported challenges in access to routines and protective equipment [19]. There were also severe challenges within home care services [20]. The mortality rates varied between different regions in the country, possibly depending on both the overall spread of Covid-19 and the organization of the elderly care [21].

The work situation for health care workers was under pressure, not only in Sweden [22], but also elsewhere during the pandemic [23, 24]. For home care workers, there have been many studies that report on a challenging work situation during the pandemic [25–27]. For example, to avoid the spread of infections different types of protective equipment and clothing were needed [26]. The pandemic also resulted in many home care recipients cancelling their services [28]. However, there is limited knowledge about how changes due to the pandemic affected the working environment in the care recipient's homes.

In the current study, our aim was to investigate the in-home work environment for home care workers by focusing on two different aspects of the work. First, we explored different situations that arise in home care regardless of whether the work takes place during a pandemic or not. Second, we investigated specific challenges due to Covid-19 in working with home care recipients. For the latter aspect, we also compared regions that were affected by Covid-19 to different extents.

## Methods

### Design

Two cross-sectional questionnaire surveys were performed, the first in 2017 and the second in 2021–2022, both using a 12-page paper questionnaire named *A study about work environment in the home care.*

### Setting

The current study was conducted in three regions in northern Sweden that varied in their mortality rate from Covid-19, including Västernorrland, which was one of the regions with the highest mortality, and Jämtland/Härjedalen and Västerbotten, which belonged to the regions with the lowest mortality [21]. The surveys were part of the WeWorC (Work Environment and Work Conditions) project, and previously published results from this project have covered questions related to home care workers’ workload and health [5, 10].

### Participants and data collection

All 30 municipalities in the three regions were invited to participate in the studies. Of them, 16 municipalities participated before the pandemic and 22 municipalities participated during the pandemic. Two municipalities only participated before the pandemic, one in Jämtland/Härjedalen and one in Västernorrland, eight municipalities only participated during the pandemic, all of them in Västerbotten. These deviations in participating municipalities between the two surveys resulted in changes in the distribution of invited participants for the regions between the two surveys.

Participating municipalities committed themselves to distribute the questionnaire to their municipal home care workers. Private providers are common in Sweden, but were not invited to the study, mainly because of logistic challenges, but also to limit the focus on the municipality home care workers. The first survey was distributed in October 2017, and the second survey was distributed from September 2021 to April 2022. We instructed each municipality’s contact person to distribute the questionnaire to staff who are part of a working group and thereby have sufficient work experience.

In total, around 2000 home care workers were given the opportunity to respond to the questionnaire before the pandemic, of whom 1154 responded (response rate 58%) [10]. In most cases before the pandemic the staff responded to questionnaires during workplace meetings, while during the pandemic the questionnaire was commonly responded to outside of work meetings, which limited our possibility to accurately estimate the numbers of home care workers invited. Our estimate is that around 1900 had the possibility to participate during the pandemic, of whom 629 people responded to the questionnaire (estimated response rate 33%). We obtained the number of employed home care workers that each municipality expected to invite to the study. We requested exact number of invited workers, but most municipalities could only confirm approximate numbers. Therefore, our numbers of invited employees are approximate but still likely to be accurate. In the current study, the respondents were asked for their birth year, which was used to calculate age using 2017 and 2021 as response years, and the country they were living in for most of the first 10 years of life, which we used as the country of birth. Among home care workers in Swedish municipalities in general, most of whom work within elderly care, 34 percent of the assistant nurses and 48 percent of the nursing aids were born outside Sweden in 2022 [29]. Due to language barriers in these groups, we expected lower participation rates among foreign-born than among Swedish-born in both surveys. Characteristics, and their response alternatives, of the study participants are presented in Table 1.

**Table 1:** Characteristics

	Before the pandemic; 2017 (*n* = 1,154)		During the pandemic; 2021/2022 (*n* = 629)	
**Gender**^a^	n	%	n	%
Man	181	16%	106	17%
Woman	964	84%	515	83%
Other	-		3	0.5%
**Country of birth**^**c**^				
Sweden	1,079	94%	566	91%
Other	69	6.0%	55	8.9%
**Housing situation**				
Alone	235	20%	138	22%
Partner	778	68%	425	68%
Living with child	91	7.9%	45	7.2%
With others	43	3.7%	17	2.7%
**Tenure**				
< 1^b^	99	8.6%	40	6.4%
1–5 years^b^	334	29%	183	29%
6–15 years	350	30%	186	30%
> 15 years	365	32%	216	35%
**Health education**				
Assistant nurse	778	68%	439	71%
Nursing assistant	138	12%	84	14%
Other health education	35	3.1%	17	2.7%
No formal health education	193	17%	82	13%
**Employment form**^**c**^				
Permanent	972	84%	572	91%
Temporary contract	84	7.3%	32	5.1%
Hourly	95	8.3%	24	3.8%
	n	Median; Quartiles	n	Median; Quartiles
**Age**^**c**^	1,135	45; 30/55	619	47; 34/57
**Working hours per week**^**c**^	1,104	37; 30/37	605	37; 31/37
**Proportion working on a team**^**c**^	1,082	16; 10/30	572	20; 10/47
**Proportion doing night work**^**c**^	1,078	8; 4/12	577	8; 5/15

^a^ “Other” excluded from the analyses

^b^The response alternatives were “less than 1½ years” and “1½-5 years” in the survey during the pandemic

^c^Statistical significance at the 5% level for comparisons between before and during the pandemic

Questions related to the in-home work environment with specified potential problems for the home care worker and how frequently they experienced these as problems were repeated in questionnaires. There was also an option to specify other problems and the extent of such problems. Additionally, Covid-19–related questions were part of the latter survey, which was the only relevant difference between the surveys for the current study. Tables 2, 3, 4 present questions covering this, and their response alternatives are presented except for the question “Are you part of a Covid-19 team at your workplace?”.

**Table 2:** Experienced problems with the in-home work environment before (*n* = 1,154) and during the pandemic (*n* = 629)

Work environment problem^a^	Several times a day		1–2 times a day		At least once a week		At least once a month		Seldom/Never		p^b^
	2017	2021	2017	2021	2017	2021	2017	2021	2017	2021	
	n(%)^c^	n (%)	n (%)	n (%)	n (%)	n (%)	n (%)	n (%)	n (%)	n (%)	
Poor bed (*n* = 1,115/599)	127 (12%)	115 (19%)	200 (18%)	108 (18%)	338 (30%)	189 (32%)	206 (18%)	107 (18%)	244 (22%)	80 (13%)	0.001
Small toilet (n = 1,121/604)	186 (17%)	163 (27%)	260 (23%)	136 (23%)	365 (33%)	153 (25%)	169 (15%)	87 (14%)	141 (13%)	65 (11%)	< 0.001
Smoking (before or during visit) (*n* = 1,132/612)	114 (10%)	90 (15%)	154 (14%)	97 (16%)	371 (33%)	190 (31%)	174 (15%)	76 (12%)	319 (28%)	159 (26%)	0.002
Pets (*n* = 1,129/607)	72 (6.4%)	74 (12%)	141 (12%)	80 (13%)	266 (24%)	165 (27%)	141 (12%)	77 (13%)	509 (45%)	211 (35%)	0.002
Cleaning equipment (*n* = 1,129/603)	23 (2.1%)	29 (4.8%)	70 (6.3%)	43 (7.1%)	275 (25%)	175 (29%)	356 (32%)	200 (33%)	395 (35%)	156 (26%)	0.016
Relatives or friends (*n* = 1,129/604)	10 (0.9%)	13 (2.1%)	37 (3.3%)	32 (5.3%)	150 (13%)	95 (16%)	292 (26%)	153 (25%)	640 (56%)	311 (51%)	0.004
Handling of cash (*n* = 1,130/602)	14 (1.2%)	4 (0.7%)	24 (2.1%)	14 (2.3%)	183 (16%)	82 (14%)	245 (22%)	106 (18%)	664 (59%)	396 (66%)	0.676
Hot or cold room temperature (*n* = 1,129/601)	115 (10%)	90 (15%)	117 (10%)	78 (13%)	228 (20%)	119 (20%)	209 (19%)	109 (18%)	460 (41%)	205 (34%)	0.001
Under the influence / intoxicated care recipient (*n* = 1,131/606)	10 (0.9%)	11 (1.8%)	21 (1.9%)	24 (4.0%)	147 (13%)	81 (13%)	253 (22%)	148 (24%)	700 (62%)	342 (56%)	0.002
Verbal harassment, e.g. racism (*n* = 1,130/606)	4 (0.4%)	7 (1.2%)	19 (1.7%)	23 (3.8%)	98 (8.7%)	71 (12%)	181 (16%)	91 (15%)	828 (73%)	414 (68%)	0.001

^a^n refers to the number of responders to the question in 2017 and 2021/2022 (during the pandemic)

^b^Comparison of daily problems, i.e. the response alternatives “Several times a day” and “1–2 times a day”, between the 2017 and 2021 surveys

^c^n refers to the number of responses for this response option, and % refers to the percentage of this response option in the given year

Responses were given to the question: “To what extent do you experience problems in your work environment in the home care recipient’s home?”

**Table 3:** Home care recipients infected by Covid-19 (*n* = 629)

**Question**	Region	None	< 10%	10–30%	30–50%	> 50%	Don’t know
*Estimate the proportion of infected home care recipients in your unit’s catchment area?*	Jämtland/Härjedalen (*n* = 79)	7 (8.9%)	41 (52%)	18 (23%)	3 (3.8%)	1 (1.3%)	9 (11%)
	Västerbotten (*n* = 177)	65 (37%)	73 (41%)	16 (9.0%)	4 (2.3%)	3 (1.7%)	16 (9%)
	Västernorrland (*n* = 362)	32 (8.8%)	180 (50%)	87 (24%)	11 (3.0%)	12 (3.3%)	40 (11%)
*Estimate the extent to which your home care recipients have chosen to forgo home care?*	Jämtland/Härjedalen (*n* = 78)	8 (10%)	34 (44%)	18 (23%)	1 (1.3%)	0	17 (22%)
	Västerbotten (*n* = 177)	30 (17%)	75 (42%)	22 (12%)	6 (3.3%)	1 (0.6%)	43 (24%)
	Västernorrland (*n* = 363)	62 (17%)	168 (46%)	51 (14%)	8 (2.2%)	2 (0.6%)	72 (20%)
		Several times a day	Few times a day	Few times a week	Sparsely	Never	
*Estimate how often you have met home care recipients with Covid-19 during high infection rates within your unit?*	Jämtland/Härjedalen (*n* = 78)	3 (3.8%)	2 (2.6%)	16 (21%)	28 (36%)	29 (37%)	
	Västerbotten (*n* = 177)	14 (7.9%)	11 (6.2%)	11 (6.2%)	38 (21%)	103 (58%)	
	Västernorrland (*n* = 362)	67 (19%)	33 (9.1%)	49 (14%)	136 (38%)	77 (21%)

**Table 4:** Home care workers perceptions of challenges during the Covid-19 pandemic (*n* = 629)

		**Very large**	**Large**	**Average**	**Small**	**Very small**
Worried about being infected during working hours*	Jämtland/Härjedalen	14 (18%)	14 (18%)	15 (19%)	21 (27%)	15 (19%)
	Västerbotten	19 (11%)	32 (18%)	37 (21%)	51 (29%)	37 (21%)
	Västernorrland	74 (20%)	110 (30%)	57 (16%)	78 (21%)	48 (13%)
Concern from home care recipients about being infected by staff*	Jämtland/Härjedalen	10 (13%)	27 (34%)	18 (23%)	16 (20%)	8 (10%)
	Västerbotten	19 (11%)	50 (29%)	42 (24%)	50 (29%)	14 (8.0%)
	Västernorrland	73 (20%)	129 (35%)	71 (19%)	71 (19%)	21 (5.8%)
Overtime/extra shifts*	Jämtland/Härjedalen	3 (3.8%)	6 (7.6%)	18 (23%)	30 (38%)	22 (28%)
	Västerbotten	20 (11%)	29 (16%)	26 (15%)	52 (30%)	49 (28%)
	Västernorrland	26 (7.2%)	45 (13%)	77 (21%)	117 (33%)	94 (26%)
Lack/no protective equipment*	Jämtland/Härjedalen	2 (2.5%)	8 (10%)	15 (19%)	29 (37%)	25 (32%)
	Västerbotten	5 (2.8%)	22 (13%)	27 (15%)	60 (34%)	62 (35%)
	Västernorrland	29 (8.0%)	66 (18%)	62 (17%)	102 (28%)	102 (28%)
Unavailable protective equipment*	Jämtland/Härjedalen	3 (3.8%)	4 (5.1%)	14 (18%)	29 (37%)	28 (36%)
	Västerbotten	4 (2.3%)	11 (6.3%)	32 (18%)	52 (30%)	77 (44%)
	Västernorrland	23 (6.4%)	57 (16%)	61 (17%)	94 (26%)	124 (38%)
Lack of skills or lack of information among coworkers about Covid-19*	Jämtland/Härjedalen	3 (3.8%)	9 (12%)	24 (31%)	23 (29%)	19 (24%)
	Västerbotten	9 (5.1%)	18 (10%)	28 (16%)	63 (36%)	57 (33%)
	Västernorrland	24 (6.7%)	49 (14%)	86 (24%)	104 (29%)	94 (26%)
Covid-19 adaptation of the workplace*	Jämtland/Härjedalen	1 (1.3%)	5 (6.7%)	25 (33%)	23 (31%)	21 (28%)
	Västerbotten	15 (8.6%)	28 (16%)	30 (17%)	39 (22%)	62 (36%)
	Västernorrland	32 (8.9%)	58 (16%)	79 (22%)	101 (28%)	89 (25%)
Use of face masks*	Jämtland/Härjedalen	10 (13%)	18 (23%)	10 (13%)	20 (26%)	20 (26%)
	Västerbotten	51 (29%)	29 (16%)	18 (10%)	28 (16%)	50 (28%)
	Västernorrland	42 (12%)	74 (21%)	60 (17%)	87 (24%)	96 (27%)
Use of other protective equipment*	Jämtland/Härjedalen	3 (3.8%)	13 (17%)	18(23%)	18 (23%)	26 (33%)
	Västerbotten	23 (13%)	30 (17%)	26 (15%)	37 (21%)	59 (34%)
	Västernorrland	53 (15%)	72 (20%)	58 (16%)	75 (21%)	98 (28%)
Difficulty socializing with home care recipients*	Jämtland/Härjedalen	9 (12%)	23 (30%)	16 (21%)	16 (21%)	13 (17%)
	Västerbotten	40 (23%)	66 (38%)	21 (12%)	28 (16%)	20 (11%)
	Västernorrland	56 (16%)	111 (31%)	81 (23%)	67 (19%)	44 (12%)

^*^Statistical significance at the 5% level, comparing the response alternatives “very large” or “large” with the response alternatives “average”, “small”, or “very small”

The question for each of the issues was: “To what extent have the following been a problem for you during the pandemic?”. The questions and response alternatives were according to the table

### Statistics

Descriptive statistics were used to present the characteristics of the sample. For the questions regarding in-home work environment, the response alternatives “Several times a day” and “1–2 times a day” were defined as daily problems, while other response alternatives were defined as “Less than once a day”. For each participant who responded to all 10 of the listed work environment problems, the number of daily reported problems was summed and the average number of problems was calculated. Student’s t-test was used to compare differences between the survey years, and Pearson’s c^2^-test was used to test for differences between regions. Trends in the average number of in-home work environment problems for home care workers were tested with two-way analysis of variance using interaction terms for survey year and region. Stata 13.1 (StataCorp, College Station, TX) was used for analyses. Statistical significance was defined at the 5% level. The results are presented for the three regions that participated.

## Results

In both surveys, the majority of the 1,783 responders were women (84%) and had an education as an assistant nurse (69%) (Table 1). There was a statistically significant difference between the two surveys in terms of age, with older participants in the survey during the pandemic, country at birth, where the percentage of foreign-born participants was higher during the pandemic, and employment form, where the percentage of permanently employed was higher during the pandemic. There were statistically significantly more working hours, working in teams, and night work among respondents during the pandemic. There was a statistically significant difference (*p* < 0.001) in the number of participants between the three participating regions for the two surveys (Table 5), with 75 percent fewer participating in the second survey in Jämtland/Härjedalen (from 330 to 81 participants), 44 percent fewer participating in the second survey in Västernorrland (from 667 to 371 participants), and 13 percent more participating in the second survey in Västerbotten (from 157 to 177 participants).

**Table 5:** In-home work environment before (*n* = 1,154) and during the pandemic (*n* = 629) divided by participating regions

**Work environment problem**	**Survey year**	**Daily problems**			**Less than once a day**		
		Jämtland/Härjedalen (*n* = 330/81)^a^	Västerbotten (*n* = 157/177)^a^	Västernorrland (*n* = 667/371)^a^	Jämtland/Härjedalen (*n* = 330/81)^a^	Västerbotten (*n* = 157/177)^a^	Västernorrland (*n*=667/371)^a^
Poor bed	2017	77 (24%)	46 (30%)	204 (32%)	240 (76%)	105 (70%)	443 (68%)
	2021^b^	18 (25%)	74 (43%)^c^	131 (37%)	54 (75%)	98 (57%)	224 (63%)
Small toilet	2017^b^	125 (38%)	39 (26%)	282 (44%)	197 (61%)	112 (74%)	366 (56%)
	2021	35 (48%)	87 (50%)^c^	177 (50%)	38 (52%)	87 (50%)	180 (50%)
Smoking (before or during visit)	2017^b^	90 (28%)	21 (14%)	157 (24%)	236 (72%)	134 (86%)	494 (76%)
	2021	17 (22%)	59 (34%)^c^	111 (31%)^c^	60 (78%)	115 (66%)	250 (69%)
Pets	2017	64(20%)	26 (17%)	123 (19%)	262 (80%)	126 (83%)	528 (81%)
	2021	16 (21%)	42 (24%)	96 (27%)^c^	60 (79%)	132 (76%)	261 (73%)
Cleaning equipment	2017	26 (8.1%)	18 (12%)	49 (7.6%)	295 (92%)	135 (88%)	596 (92%)
	2021^b^	9 (12%)	31 (18%)	32 (9.1%)	66 (88%)	146 (82%)	319 (91%)
Relatives or friends	2017	16 (4.9%)	5 (3.2%)	26 (4.0%)	308 (95%)	149 (97%)	625 (96%)
	2021	6 (8.0%)	12 (6.9%)	27 (7.6%)^c^	69 (92%)	163 (93%)	327 (92%)
Handling of cash	2017	10 (3.1%)	5 (3.3%)	23 (3.5%)	314 (97%)	148 (97%)	630 (96%)
	2021	0	9 (5.1%)	9 (2.6%)	74 (100%)	167 (95%)	343 (97%)
Hot or cold room temperature	2017	68 (21%)	34 (22%)	130 (20%)	256 (79%)	120 (78%)	521 (80%)
	2021	17 (23%)	59 (34%)^c^	92 (26%)^c^	57 (77%)	116 (66%)	260 (74%)
Under the influence / intoxicated care recipient	2017	7 (2.2%)	2 (1.3%)	22 (3.4%)	317 (98%)	151 (99%)	632 (97%)
	2021^b^	1 (1.3%)	17 (9.7%)^c^	17 (4.8%)	75 (99%)	158 (90%)	338 (95%)
Verbal harassment, e.g. racism	2017	6 (1.9%)	1 (0.6%)	16 (2.4%)	318 (98%)	153 (99%)	636 (98%)
	2021	4 (5.4%)	7 (4.0%)^d^	19 (5.3%)^c^	70 (95%)	168 (96%)	338 (95%)
Total; average^e^	2017	489; 1.48	197; 1.33	1032; 1.56			
	2021	123; 1.66	397; 2.21^c^	711; 1.94^c^			

^a^Number of participants in each region before and during the pandemic

^b^Statistical significance when comparing the occurrence of experienced daily problems between regions during the survey year

^c^Statistical significance when comparing changes in experienced daily problems in the region before and during the pandemic

^d^Statistical significance but violating assumptions for ${\chi }^{2}$ test due to too low expected counts

^e^In the last row, the number of reported daily problems for any of the problems listed are summed, and thereafter the average number of problems per home care worker is reported among those responding to all of the questions. No statistical tests were performed to compare between years or between regions

### In-home work environment problems

Frequent problems in the work environment were reported by a high percentage of the participating home care workers before the pandemic (Table 2). During the pandemic, the problems in the daily work environment were exacerbated, with a statistically significant increase for all listed problems except for “handling of cash” (Supplementary Table 1). Problems were increased from before to during the pandemic for impractical bathrooms (40% vs. 50%), non-ergonomic beds (29% vs. 37%), indoor smoking (24% vs. 31%), excessively high or low temperatures in the homes (21% vs. 28%), and pets (19% vs. 25%). Furthermore, as many as 11% of the employees had experienced verbal harassments, for example racist remarks, at least once a week before the pandemic, and this increased to 17% during the pandemic. In addition to the experienced work environment problems presented in Table 2 based on pre-set response options, other problems were mentioned by 69 respondents, including sexual harassment (*n* = 11), threatening or violent behaviour (*n* = 7), abusive behaviour (*n* = 5), and time constraints (*n* = 5).

### Work environment problems by region

Before the pandemic, there were statistically significant variations in daily experiences of problems between regions for impractical bathrooms, ranging from 26 to 44%, and indoor smoking, ranging from 14 to 28% (Table 5). During the pandemic, there were statistically significant differences between regions for non-ergonomic beds, ranging from 25 to 43%, poor cleaning equipment, ranging from 9.1% to 18%, and intoxicated care recipients, ranging from 1.3% to 9.7%. There were also some statistically significant differences in the reporting of work environment problems between the regions. The average number of reported in-home work environment problems showed a statistically significant increase during the pandemic in Västerbotten, from 1.33 to 2.21 problems, and in Västernorrland, from 1.56 to 1.94 problems, while there was a non-statistically significant increase from 1.48 to 1.66 problems in Jämtland/Härjedalen. Thus, the lowest average number of problems were reported in Västerbotten before the pandemic, and this increased to be the highest reported average number of problems during the pandemic. However, while the descriptive results indicate a trend in the average number of experienced work environment problems shifting between the regions, there was no statistically significant increase in the number of problems from before to during the pandemic.

### Challenges related to Covid-19

There were some infected home care recipients in all regions, being more common in Jämtland/Härjedalen and Västernorrland than in Västerbotten. Home care workers in Västernorrland (28%) met infected home care recipients more frequently on a daily basis during high infection rates than in Jämtland/Härjedalen (6.4%) and Västerbotten (14%) (Table 3). Most (62%) thought that there were home care recipients who had cancelled their home care services due to Covid-19, but only to a limited degree (less than 3% estimated it to be more than 30% of the home care recipients). A total of 45 (7.3%) of the respondents reported that they were part of a Covid-19 work team.

Home care workers expressed worries about being infected themselves (42%) and concern from home care recipients about being infected (50%) (Table 4). There were statistically significant differences between the regions in terms of worries from the staff themselves and from the home care recipients about being infected, with the greatest worries in Västernorrland. Some of the staff reported challenges with working overtime (21%), a lack of protective equipment (21%), inaccessible protective equipment (17%), and a lack of competence among coworkers (18%). There were statistically significant differences in problems due to inaccessible and lack of protective equipment between the regions. For these issues, Västernorrland had the biggest challenges with 22 and 26% reporting major problems with them compared to 9 and 15% in Västerbotten, and 9 and 13% in Jämtland/Härjedalen. The adaptation of the workplace to Covid-19 was well managed in Jämtland/Härjedalen (only 6 of 77 considering it as a problem), while there were statistically significantly more problems reported from the other two regions (25% of the responders in both regions). The use of face masks was reported to be a challenge by 37% of the home care workers, and issues with other equipment were reported by a slightly lower percentage of the participants (32%), with statistically significant variations between the three regions. Around half of the home care workers reported major challenges in socializing with home care recipients during the pandemic with a statistically significant variation between regions, with most problems reported in Västerbotten (71% of the staff).

## Discussion

The results from our two cross-sectional studies on the work environment among home care workers in northern Sweden showed that staff regularly experienced problems with the in-home work environment prior to the pandemic and that work environment problems were more frequently reported during the pandemic. On a daily basis, challenges with impractical bathrooms, non-ergonomic beds, indoor smoking, in-home temperature, and pets were most commonly reported, but also problems with verbal harassment were reported. Regional differences in the reporting of in-home work environment problems were seen, which implies that different priorities might be advisable for regions as well as for municipalities, at least under normal conditions such as before the pandemic. Our interpretation is that levels of problems both before and during the pandemic can be considered high for most occupational health challenges in all regions. Our study also presents pandemic-specific challenges that varied between regions. Worries about infections and the use of face masks were frequent problems among home care workers, as well as challenges with social interactions that might be connected to the use of safety equipment.

Little research has investigated the in-home work environment for home care workers. In a Norwegian study prior to the pandemic, inappropriate verbal interactions with the home care recipients and environmental hazards and unhealthy physical workloads were part of the key results [1]. Importantly, our reported challenges were not a consequence of the pandemic, but were common already prior to the pandemic. The pandemic brought attention to things that are particularly vulnerable when resources become scarcer. While our results are based on two cross sectional surveys, which limit the possibility to draw causal conclusions, our study suggests that with more strained working conditions the work situation becomes more challenging for home care workers.

However, it is not easy to interpret which in-home work environment problems are perceived as the most severe for the home care workers based on our survey because we only asked whether they experienced a problem and how often and not the burden of the problem on each occasion. Thus, the less frequently occurring harassments, which are a psychosocial problem for the home care workers, might overall be more demanding than the more frequently appearing ergonomic problems such as non-ergonomic beds and impractical bathrooms. We did not specify the type of harassments experienced by the staff. Challenges with sexual harassment among health care workers has been reported before and have been summarized in a recent review [30], but responses in our case could also relate to racism and other harassments. Despite harassment occurring in Swedish home care, the extent of such harassment is poorly documented. Future studies that can better explain the extent of in-home work environment problems and suggest improvements are important because such scientific information is lacking.

According to information we have received, we expect that among existing home care recipients, somewhere between 30 to 40% have received their first home care support during the year. During the pandemic, the municipalities likely updated the care recipients’ homes to a lesser extent, both to avoid the risk of an increased level of Covid-19 among them, but also due to less resources to make the necessary changes. We believe this is the main explanation for an increase in ergonomic problems during the pandemic.

There was also an increase in problems due to poor cleaning equipment during the pandemic. We expect that this is mainly because home care recipients did not update their equipment as much during the pandemic because they did not visit stores as often due to concerns about being infected. The home care workers also reported problems related to the behaviour of the home care recipient, as some were smoking, there were verbal harassments and there also was problems with the recipient being under the influence. These problems increased during the pandemic, which potentially might be explained by them being more isolated due to the risk of spreading Covid-19 increased.

Under certain circumstances it is possible to deny people home care and instead offer them nursing homes due to high costs according to the Social Services Act [3]. However, to our understanding people cannot be denied home care due to work environment problems for home care workers, despite the fact that Swedish work environment legislation demands that the employees have a suitable work environment [4]. To handle this, employers can redistribute the staff caring for home care recipients, such as having home care workers with allergies avoiding contact with pets, but can also try to have a dialogue with the home care recipient and their relatives. Dialogue models have been tested in the rehabilitation of patients with dementia in Sweden [31], and in such dialogues different professions collaborate with the care recipient and their relatives. Ways forward are needed to handle the conflicting challenge between the home care recipients' autonomy and the home care workers’ work environment. For ergonomic problems, such as non-ergonomic beds and impractical bathrooms, potential solutions are likely simpler than for issues such as harassment, smoking, and pets. There are challenges in recruiting staff to the home care services, and it is common that staff want to leave the occupation [16, 17], and thus a well-working in-home work environment certainly must be of high importance [6].

During the pandemic, there were variations in the preparedness between different regions in Sweden [32], and the rates of infections and deaths varied [21]. We consider our results to well illustrate such differences between three northern regions, where Västernorrland was one of the regions with the greatest challenges during the pandemic and Västerbotten and Jämtland/Härjedalen were among the regions with the least challenges. For instance, the availability of and use of protective equipment was better in Västerbotten than in Västernorrland, and the least concern about being infected among home care workers and home care recipients was in Västerbotten. Interestingly, it appears that the regions with the lowest infection rates had as a side effect that social interactions with home care recipients were limited. In a previous study from our survey before the pandemic, more time for social support was asked for by home care workers [5]. Because social interaction is limited when the staff use protective equipment, such as face masks, better solutions for maintaining well-functioning social interaction with home care recipients despite the use of protective equipment can therefore be recommended.

Our point prevalences for both of the surveys should also be understood based on who responded to the surveys. Due to problems with recruiting staff, it is common among home care workers to have limited knowledge of Swedish, even though the Swedish language is a stated requirement for employment. The proportion of foreign-born responders in our study was much lower than the proportion of foreign-born home care workers reported by the Swedish Association of Local Authorities and Regions for Sweden [29]. Thus, we expect there to be a low response rate among foreign-born responders in our study. Translating the surveys into other languages to include their participation would have required more resources than we deemed reasonable, both for the research group and the home care organizations. If we would have been able to increase their participation in our surveys, our results likely would be affected. Thus, our results might mainly reflect the situation for Swedish-born home care workers, and a further follow-up of migrant workers would be valuable.

There was variation in the participating municipalities and a lower participation rate among home care workers during the pandemic. There was also a variation in terms of who answered the questionnaire, such as more night staff and more foreign-born responding during the pandemic. A lower participation rate during the pandemic might mean that there was a variation in who wanted to participate in the surveys. For instance, staff who had a less challenging situation during the pandemic, but also during “normal” circumstances, might have participated to a higher extent, as well as staff with a more negative view. Besides the limitations related to variation in the characteristics of who responded to the surveys, the low response rate in itself could also affect our overall conclusions. Even if the participants likely varied to some extent between the surveys, and there might be a bias in the prevalence of the extent of problems, we nevertheless expect the extent of the problems both before and during the pandemic to well represent the current situation.

The northern part of Sweden differs from the rest of the country, for instance in population density, which could limit the generalizability of our results to other parts of the country. However, despite some differences in challenges due to the colder climate and longer travel distances in the more sparsely populated Northern part of Sweden that we have included in our study, we do not expect the work environment-related problems we have investigated to differ notably in other regions in Sweden. Our study was limited to municipal home care providers. We expect similar challenges, but they may differ somewhat, among private providers and that study results would therefore be very valuable for them as well. In addition, the organization of home care in Sweden differs from many other countries, and there could therefore be limitations in the generalizability to other countries. However, the interaction between home care workers and home care recipients is inevitably part of the work in all countries, and we therefore expect our study results to also be of relevance outside Sweden, especially in countries with similar arrangements for their home care services.

In our studies for the WeWorC project we have also collected data about home care workers’ health, and it would have been valuable to investigate whether a poor working environment for home care workers is linked to health challenges. We have also collected narratives within the surveys from the home care workers about their work situation that could help with interpretations of the survey results. These are topics that we aim to evaluate in future research within the WeWorC project.

The pandemic challenged the organization within Swedish home care. The use of and availability of protective equipment have improved and a learning lesson from the pandemic is that staff are more aware of using them to protect home care recipients. Our study adds valuable knowledge about challenges that will be useful to the organizations even after the pandemic. To our knowledge, there are no studies that have investigated whether the use of and availability of protective equipment works well after the pandemic.

## Conclusions

Our study highlights a topic where little research has been conducted. Through our study we can inform the home care organizations and the responsible governmental organizations about the extent of various problems in order to help them decide on prioritizations. Normally, laws can regulate work environments and improve such environments for employees, which, for instance, has been done through regulations against smoking in restaurants in many countries [33]. However, limiting people in their own homes is controversial. Solutions to the work environment-related problems caused by the behavior of home care recipients that were expressed by the home care staff in our study are therefore not easy to solve. However, we also identified physical limitations in providing services to home care recipients. These have a more obvious space for improvements for the home care workers, although any changes in the home care recipients’ homes needs must be agreed upon.

## Supplementary Information

Supplementary Material 1.

## Data Availability

The datasets generated and/or analysed during the current study are not publicly available because the Swedish Data Protection Act (1998:204) does not permit sensitive data on humans to be freely shared. The datasets are available based on ethical permission from the Regional Ethical board in Umeå, Sweden, from one of the authors (Fredrik Norström).
