# Supplementary material for: In-home work environment for home care workers in Northern Sweden before and during the Covid-19 pandemic
Source: BMC Health Serv Res. 2025 Jan 24;25:137. doi: 10.1186/s12913-024-12161-y (PMC11760649; doi:10.1186/s12913-024-12161-y)
Supplement: Supplementary file 1 — Supplementary Material 1. [file 12913_2024_12161_MOESM1_ESM.docx]

**Supplementary Table 1.** Experienced problems with the in-home work environment before (n = 1,154) and during the pandemic (n = 629).

| **Work environment problem^a^** | **Daily^b^** | | | | **Not daily** | | | |  |
| --- | --- | --- | --- | --- | --- | --- | --- | --- | --- |
|  | 2017 | | 2021 | | 2017 | | 2021 | |  |
|  | n | % | n | % | n | % | n | % | **P^c^** |
| Poor bed (n = 1,115/599) | 327 | 29% | 223 | 37% | 788 | 71% | 376 | 63% | 0.001 |
| Small toilet (n = 1,121/604) | 446 | 40% | 299 | 50% | 675 | 60% | 305 | 50% | <0.001 |
| Smoking (before or during visit) (n = 1,132/612) | 268 | 24% | 187 | 31% | 864 | 76% | 425 | 69% | 0.002 |
| Pets (n = 1,129/607) | 213 | 19% | 154 | 25% | 916 | 81% | 453 | 75% | 0.002 |
| Cleaning equipment (n = 1,129/603) | 93 | 8.3% | 72 | 12% | 1,026 | 92% | 531 | 88% | 0.016 |
| Relatives or friends (n = 1,129/604) | 47 | 4.2% | 45 | 7.5% | 1,082 | 96% | 559 | 93% | 0.004 |
| Handling of cash (n = 1,130/602) | 38 | 3.4% | 18 | 3.0% | 1,092 | 97% | 584 | 97% | 0.676 |
| Hot or cold room temperature (n = 1,129/601) | 232 | 21% | 168 | 28% | 897 | 79% | 433 | 72% | 0.001 |
| Under the influence / intoxicated care recipient (n = 1,131/606) | 31 | 2.7% | 35 | 5.8% | 1,100 | 97% | 571 | 94% | 0.002 |
| Verbal harassment, e.g. racism (n = 1,130/606) | 23 | 2.0% | 30 | 5.0% | 1,107 | 98% | 576 | 95% | 0.001 |

^a^ n refers to the number of responders to the question in 2017 and 2021/2022 (during the pandemic).
^b^ n refers to the number of responses for this response option, and % refers to the percentage of this response option in the given year.
^c^ Comparison of daily problems, i.e. the response alternatives “Several times a day” and “1-2 times a day”, between the 2017 and 2021 surveys.

Responses were given to the question: “To what extent do you experience problems in your work environment in the home care recipient’s home?”
